# Use of family disability service by families with young children with disabilities

**DOI:** 10.1111/dmcn.14478

**Published:** 2020-01-31

**Authors:** Matthew J Russell, Yunqi Zhang, Xinjie Cui, Suzanne Tough, Jennifer D Zwicker

**Affiliations:** ^1^ PolicyWise for Children & Families Edmonton Canada; ^2^ Community Health Sciences Cumming School of Medicine University of Calgary Calgary Canada; ^3^ School of Public Policy University of Calgary Calgary Canada; ^4^ Paediatrics Cumming School of Medicine University of Calgary Calgary Canada; ^5^ Department of Kinesiology University of Calgary Calgary Canada

## Abstract

**Aim:**

To investigate which families with young children with disabilities used disability services and when they used services to inform policy on service delivery.

**Method:**

We used linked administrative data from different ministries in Alberta to describe families’ use of disability services when their children were between the ages of 3 and 8 years old. Disability was investigated on the basis of the presence of a severe special education code for children, and level of special education code. The outcome was the use of family disability services.

**Results:**

Of 31 346 children, 24 761 (79.0%) had no special education code, 3982 (12.7%) had a mild special education code, and 2603 (8.3%) had a severe special education code. Level of special education code was associated with child characteristics and service use. Children with severe special education codes generally were more likely to report service use and have poor outcomes than those with less severe codes. Of note, 26% of children with severe special education codes used family disability services. In addition, among children with severe special education codes, many years of severe coding (compared with fewer years) had the strongest association with family disability service use (prevalence ratio 5.50; 95% confidence interval 4.10–7.37). Associations with family disability service use were seen with mental health, health care, and educational achievement. Interactions between child characteristics and service use were observed.

**Interpretation:**

This study provides evidence that families were more likely to use disability services when they were involved with other services, and that use interacts with various factors. The findings highlight the importance of considering service eligibility, referral, and integration.

## Abstract 2

This article is commented on by Gubbels on pages 10 of this issue.

**Abbreviations**
FSCD: Family Support for Children with Disability
SES: Socioeconomic status

**What this paper adds**

- Children with severe special education codes were more likely to use services than those with less severe codes.
- They were also more likely to have poor outcomes than those with less severe codes.
- Fewer families used disability services early (age 3y) compared with later on.
- Families’ use of disability services corresponded with other service use.
- The study elucidated potential barriers and facilitators to disability service use.

(Signatories) recognize the equal right of all persons with disabilities to live in the community, with choices equal to others, and shall take effective and appropriate measures to facilitate full enjoyment by persons with disabilities of this right and their full inclusion and participation in the community. United Nations Convention on the Rights of Persons with Disabilities 1

For children with disabilities, full inclusion and participation in the community is often not yet a reality. As a signatory to the United Nations Convention on the Rights of Persons with Disabilities and its Optional Protocol, Canada has made a commitment to improve this situation, including collecting more data on and providing better access to supports for children with disabilities and their families.^1^ This study investigated use of the provincial family disability services in Alberta, which are services that can help facilitate the inclusion and participation of children with disabilities and support their families in line with federal United Nations Convention on the Rights of Persons with Disabilities commitments (articles 7, 23, 24, and 26).^2^, ^3^

Fifteen per cent of children and young people (2–20y) in the Alberta school system required education disability support in 2017.^4^ Children with disabilities are also more likely to have greater healthcare needs (e.g. more physician visits and more hospitalizations),^5^ mental health issues,^6^ and poor educational achievement^7^ than children without a disability. Caregivers of children with a disability often experience challenges, higher financial burden to support their child,^8^ lower quality of life^9^ and higher levels of stress, feelings of isolation and frustration, and physical and mental health issues^10^, ^11^, ^12^, ^13^ than families without children with disabilities. As disability services can help facilitate the full inclusion and participation of children with disabilities and support their families,^3^ it is important to know more about how these services are being accessed. This study used administrative data to provide information on which families with children with disabilities used ‘family disability services’ aimed at supporting the needs of children with disabilities and their families.

The early years are considered important to the developmental trajectories of children with disabilities.^14^, ^15^, ^16^ Receiving early access to support can provide a foundation to addressing barriers related to children with disabilities’ full participation in society and improve developmental trajectories.^14^, ^17^ Resources allocated to early services are thought to have a greater impact across the lifespan than later investments.^16^, ^18^, ^19^ This study investigated the use of family disability services in the early years of development for children with disabilities.

The Anderson Healthcare Utilization model proposes that three dynamic factors relate to families’ use of services: predisposing factors (sociocultural factors), enabling factors (logistical aspects of accessing care), and need.^20^ The social determinants of health, such as sex, socioeconomic status (SES), and geographical location are often related to predisposing and enabling factors.^21^ For example, SES can reduce funds available to families, limiting the options they have to pursue support services for their child, and location can affect availability of services to families.^21^ In addition, both perceived need (i.e. if parents feel their child has need) and evaluated need (i.e. if service providers state there is a need) can affect how families use services.^20^ This study investigated how potential barriers and facilitators (child characteristics and service use patterns) related to families’ use of disability services.

We used administrative data to investigate which Albertan families of children with disabilities, as identified by special education service use, made use of provincial family disability services in children’s early years of development (3–8y). We investigated when families accessed disability services, which types of disability the children had when their families accessed services, and barriers and facilitators to service access.

## Method

This study was approved through the Health Research Ethics Board of Alberta (number 1214) and the Conjoint Faculties Research Ethics Board from the University of Calgary (REB 18‐1633).

### Data set

We used administrative data from across the Child and Youth Data Laboratory. The Laboratory was a joint initiative between PolicyWise for Children & Families and six participating ministries in the Government of Alberta (Children’s Services, Community and Social Services, Health, Education, Advanced Education, and the Justice and Solicitor General). The mandate of the Child and Youth Data Laboratory was to link and analyse administrative data to provide evidence for policy and program development for the support of children and young people in Alberta. The Laboratory was linked using privacy‐preserving techniques and contained program and service use information for over 2 million children and young adults (0–30y) across Alberta from 2005/06 to 2010/11.^3^ Furthermore, as this initiative partnered with the ministries, data derivations explained below reflect stakeholder engagement and feedback to maximize their relevance to actual program use.

### Study population

For our target study population, we selected Albertan children who met three criteria: (1) they were 3 years old at the beginning of the Albertan school year in 2005/06 (and 8y in 2010/11); (2) they had at least 1 year of school registration between 2005/06 to 2010/11; and (3) they were full‐time registered in the Alberta Health Care Registry over the 6 years. This resulted in a sample of 31 346 children. Full‐time health registration was necessary to ensure that children had residence in Alberta for the duration of the study.

### Outcome: family disability service use

For our outcome, we investigated whether children’s families used disability services through the Family Support for Children with Disability (FSCD) program. The FSCD program works in partnership with eligible families to provide support that is based on their child’s and family’s assessed needs.^22^ FSCD has a variety of services: information or referral to supports, family‐focused support (e.g. cost reimbursement, counselling, or respite), or child‐focused support (e.g. aids, rehabilitation, or temporary out‐of‐home placement). FSCD services are designed to strengthen families’ ability to promote their child’s healthy development and participation in society. Outcomes were based on families’ FSCD use over the 6‐year period.

### Exposure: special education codes

To describe our population, children were defined by their special education support use, as defined by special education coding in any of the study years: no code (not coded as requiring educational support), mild code (coded for low or moderate levels of support), or severe code (coded for high levels of support).^23^ As multiple codes were possible each year and coding could change over time, we determined special education support use by the most severe code found at least once over the 6 years. Our use of the most severe code was based on Alberta Education data practices.

As most mild or not coded children were not eligible for the outcome (FSCD use), analyses focused on children who were coded as severe, with exposure based on the number of years children had a severe special education code.^22^ We compared children who had 1 to 3 years of severe special education coding with children who had 4 to 6 years of severe coding. In addition, we split special education codes by category of code (e.g. emotional/behavioural disability, multiple disability, etc.). Because codes changed over time, it was possible for children to be classified in more than one category. See descriptions of the categories in Appendix S1 (online supporting information).

### Covariates

To determine the factors underlying a family’s use of FSCD services, we investigated how child characteristics (e.g. sex and city size) and service use (e.g. mental health service use and educational achievement) related to FSCD use. Covariates were chosen on the basis of their potential impact on support access, and to children with disability and their families.^3^, ^5^, ^6^, ^7^, ^20^, ^21^ Covariates are described in detail in Appendix S1.

#### Covariates calculated over 2005/06 to 2010/11

Six covariates over 2005/06 to 2010/11 were calculated as follows: (1) Sex was based on the most commonly reported value for the child (female or male). (2) City size was based on the average population of the city where the child lived (rural<10 000; urban≥10 000). (3) SES was based on the average social and material indicators of the child’s neighbourhood (low SES, bottom 40% of Albertan neighbourhoods; high SES, top 60%). (4) Use of English as a second language was based on any program use for the child. (5) Mental health service use was based on any mental health‐related use found in health care records for the child. (6) High‐cost health care use was based on the child being in the top 5% of estimated costs for their age group and sex in any year.

#### Covariate calculated in 2010/11 only

Educational achievement was based on the child performing below expectations (having a moderate or severe intellectual disability, or severe multiple disability; unsatisfactory Provincial Achievement Test scores; or being behind one grade on the basis of their age) or meeting expectations (a lack of any of the above criteria).

### Data analysis

SAS Enterprise Guide 7.1 (SAS Institute Inc., Cary, NC, USA) was used for all statistical analyses. Descriptive statistics were used to describe the sample on the basis of child characteristics and services used for the full sample and by special education support use (no code, mild code, or severe code), and are reported as frequencies and proportions. We describe the children with mild and severe coding in relation to the general population.

**Figure 1:**
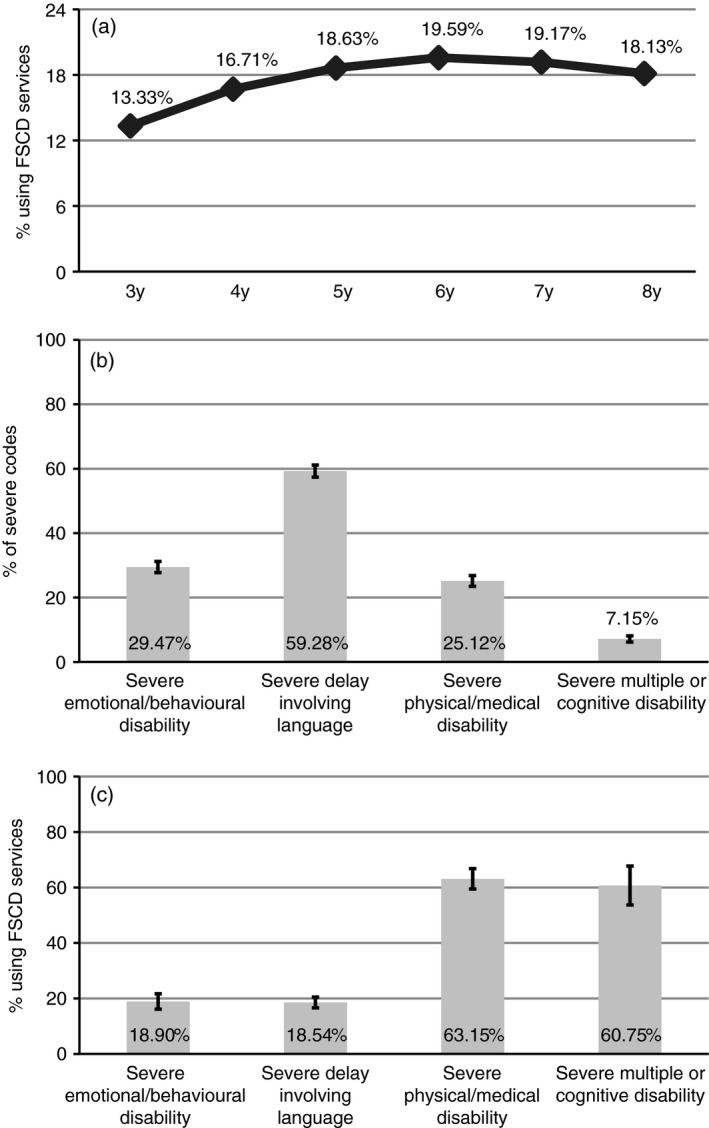
Among children with a severe special education code (a) the percentage that used Family Support for Children with Disability (FSCD) services at the listed ages and (b) the percentage that ever had the listed category of special education code. Among children’s category of special education code (c) the percentage that used FSCD services. Ninety‐five per cent confidence intervals are shown as error bars.

Next, descriptive analyses were used to report when children used FSCD services, which special education disability categories made up severe codes, and how much children with different severe special education categories used FSCD services (Fig. 1).

**Figure 2:**
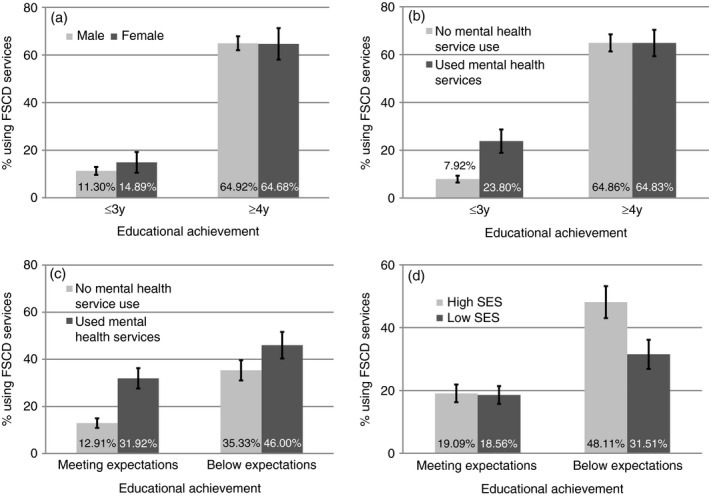
Significant interactions for the use of Family Support for Children with Disability (FSCD) services for children who had a severe special education code. The percentage of families using FSCD services for interactions between (a) years with a severe education code by sex, (b) years with a severe education code by mental health service use, (c) educational achievement by mental health service use, and (d) educational achievement by socioeconomic status (SES). Ninety‐five per cent confidence intervals are shown as error bars.

Finally, multivariable regression modelling (using a robust log‐Poisson method) was used to estimate how the length of severe coding and other covariates related to any FSCD use over the 6 years (0=no use vs 1=any use), as a prevalence ratio (Table S1, online supporting information and Fig. 2).^24^ We used log‐Poisson models for consistency, because many log‐binomial models failed to converge. While all child characteristic and service use covariates were included in the multivariable model to control for the influences of other variables, a stepwise backward approach was used to select two‐way interactions between variables that were significant (*p*<0.05). We report frequencies, proportions, and unadjusted models to show how unadjusted variables and interactions related to FSCD use. Individuals with data missing in any variable across all 6 years were not included in models (14.5%; missing data were in general more likely to be rural [39.1% vs 20.3%] and low SES [47.3% vs 38.5%]).

## Results

### Child characteristics and service use

Of 31 346 children who were aged 3 years in 2005/06, 24 761 (81.7%) had no code, 3982 (10.1%) had a mild code, and 2603 (8.3%) had a severe code. As FSCD services were mostly limited (owing to eligibility) to children with severe special education codes, the main focus of our analysis was the 2603 children coded as severe.

The full sample (all children in the cohort) showed a near‐even split on sex, with most children living in urban settings (78%). Twelve per cent had English as a second language, 10% used mental health services, and 19% used high‐cost health care services at least once over the 6 years. However, only 2.5% used FSCD services. Educational achievement was below expectations for 11% of the full sample (Table 1).

**Table 1:** Child characteristics and service use for the full sample

Child characteristics and service use	Full sample, *n*=31 346	Special education support use		
		No code, *n*=24 761	Mild code, *n*=3982	Severe code, *n*=2603
Sex				
Female	15 480 (49.38)	13 185 (53.25)	1523 (38.25)	772 (29.66)
Male	15 866 (50.62)	11 576 (46.75)	2459 (61.75)	1831 (70.34)
Missing	0 (0)	0 (0)	0 (0)	0 (0)
City size				
Rural	6896 (22.00)	5398 (21.80)	978 (24.56)	520 (19.98)
Urban	24 450 (78.00)	19 363 (78.20)	3004 (75.44)	2083 (80.02)
Missing	0 (0)	0 (0)	0 (0)	0 (0)
SES				
Low SES	12 007 (38.30)	9084 (36.69)	1638 (41.14)	1285 (49.37)
High SES	18 680 (59.60)	15 155 (61.21)	2259 (56.73)	1266 (48.63)
Missing	659 (2.1)	522 (2.1)	85 (2.13)	52 (2.0)
Student of English as a second language				
Yes	3912 (12.48)	3274 (13.22)	365 (9.17)	273 (10.49)
No	27 434 (87.52)	21 487 (86.78)	3617 (90.83)	2330 (89.51)
Missing	0 (0)	0 (0)	0 (0)	0 (0)
Mental health services				
Yes	3040 (9.70)	1714 (6.92)	494 (12.41)	832 (31.96)
No	28 306 (90.30)	23 047 (93.08)	3488 (87.59)	1771 (68.04)
Missing	0 (0)	0 (0)	0 (0)	0 (0)
High‐cost health care				
Yes	5859 (18.69)	3323 (13.42)	1156 (29.03)	1380 (53.02)
No	25 487 (81.31)	21 438 (86.58)	2826 (70.97)	1223 (46.98)
Missing	0 (0)	0 (0)	0 (0)	0 (0)
Educational achievement				
Below expectations	3092 (10.65)	1826 (7.89)	499 (13.71)	767 (33.92)
Meeting expectations	25 953 (89.35)	21 317 (92.11)	3142 (86.29)	1494 (66.08)
Missing	2301 (7.34)	1618 (6.53)	341 (8.56)	342 (13.13)
Outcome: family support for children with disabilities use				
Yes	787 (2.51)	65 (0.26)	54 (1.36)	668 (25.66)
No	30 559 (97.49)	24 696 (99.74)	3928 (98.64)	1935 (74.34)
Missing	0 (0)	0 (0)	0 (0)	0 (0)

Data are *n* (%) unless otherwise stated. Results are for all children in the cohort who were 3 years old in 2005/06 and by children’s special education support use. Percentages are summed vertically across factors (e.g. the sum of male and female). SES, socioeconomic status.

Compared with the full sample, children with severe special education codes were more likely to be male (70%) and have low SES (50%; vs 39% in the full sample). Children with severe special education codes were more likely to use mental health services (32%), be high‐cost health care users (53%), and use FSCD (26%). They were also more likely to be below expectations in educational achievement (34%). The strength of these patterns (compared with the full sample) was attenuated for mild (compared with severe) codes. Children with any special education codes were less likely to use English as a second language than those with no code (13% not coded; 9% mild; 10% severe; Table 1).

### The ‘when’ and ‘who’ of family disability service use

Among children who ever had a severe special education code, 13% used services at 3 years old, which gradually increased to 20% at 6 years and was 18% at 8 years (Fig. 1). Of the children with severe codes, most had severe delays involving language (59%), followed by emotional/behavioural (29%), physical/medical (25%), and multiple or cognitive disability (7%; see Appendix S1 for category descriptions; Fig. 1). Furthermore, the use of FSCD services was 63% for children with a severe physical/medical disability, 61% for severe multiple or cognitive disability, 19% for severe emotional/behavioural disability, and 19% for a severe delay involving language (Fig. 1). Notably, the least prevalent disabilities had the highest percentage of FSCD use, compared with the most prevalent disabilities.

### Barriers and facilitators of family disability service use

This analysis provided evidence that many factors related to the use of FSCD services (Table S1; multivariable prevalence ratios are reported below; combined fit, quasi‐likelihood information criterion 3208). Families of children with severe codes who used mental health services (prevalence ratio 3.06, 95% confidence interval [CI] 2.34–4.00), were high‐cost health care users (prevalence ratio 1.57, 95% CI 1.37–1.80), or who had many years of severe coding (≥4y; prevalence ratio 5.50, 95% CI 4.10–7.37) were significantly more likely to use FSCD services than families with children who did not use these services (adjusted). Families with children below expectations in school were also more likely to use FSCD than those meeting expectations (prevalence ratio 1.70, 95% CI 1.38–2.09). That educational achievement was related to FSCD use may relate to the complex needs that children with disabilities face, including needs that are not related to academic ability.

Finally, we found significant interactions between factors (child characteristics and service use patterns). In particular, while families with males with few years of severe special education coding (1–3y) were less likely to use FSCD than families with females, children with many years of severe coding (≥4y) did not show the same sex difference in FSCD use. Second, children who were both below expectations and had mental health visits were more likely to use FSCD services than those with either factor alone. Third, while FSCD use for families with children who met expectations did not differ by SES, among families with children below expectations, 32% with low SES and 48% with high SES used FSCD (Fig. 2).

## Discussion

As a signatory to the United Nations Convention on the Rights of Persons with Disabilities, Canada has committed to removing barriers that prevent participation by children with disabilities and their families. In Canada, many of these commitments are met through provincial service provision. In this study, we examined the family disability services provided by the Government of Alberta for children with disabilities and their families. Using cross‐ministry administrative data, we identified that children with severe special education codes were more likely to be male, live in low SES neighbourhoods, and require support (e.g. mental health services, high‐cost health care services) than the general population. This replicates demographic and health service use patterns highlighted in previous research.^25^ In addition, we found evidence that severity of special education support use related to these patterns.

Next, we found that family disability service use was often limited to children with severe special education codes, compared with those having mild coding or who were not coded. This can be interpreted in the context of eligibility, as both severe special education codes and family disability services require formal diagnoses.^22^, ^23^ As both services require diagnoses, facilitating early identification and diagnosis is important to children’s support access. In addition, it is somewhat concerning that 26% of all families with children with severe codes (whom we anticipate would be more likely to be eligible) used family disability services over the period when their child was 3 to 8 years old. While we should expect that family disability services would not be used by all, as access is voluntary,^22^ some of the remaining families may have unmet needs that would be alleviated through support. However, future research is necessary to understand the extent to which families have unmet needs, what barriers they have to accessing services, and what might improve their navigation of these services.

In addition, we described when family disability services were accessed and which children used these services. Services were less likely to be accessed at 3 years of age compared with older ages. Similar patterns have been noted for early intensive autism interventions in Ontario, with 75% of children beginning interventions at 6 years old.^19^ This is problematic, as some interventions are thought to be most effective early in life.^18^, ^19^ This study’s finding of less use of FSCD services among children of preschool age is concerning as the FSCD program facilitates access to specialists who can provide interventions.^22^ We also found that disability services were more frequently used by families with children with certain disabilities (e.g. autism, fetal alcohol spectrum disorder, intellectual disability, etc.). This may be based on need; however, some services might also benefit families with children with other disabilities. For example, therapy is thought to benefit children with emotional and behavioural issues and their caregiving families.^26^, ^27^

Several facilitators or barriers to the use of family disability services were identified. The number of years of severe special education coding was a key factor in FSCD service use; families with children who had more years of coding were more likely to use FSCD services than those with fewer years of coding. The Anderson Healthcare Utilization model suggests that need may be related to this increased use of FSCD services (e.g. they may perceive more need or be more likely to have a referral over more years).^20^ Similarly, other service needs (e.g. mental health service use, etc.) and poor educational achievement were connected to FSCD service use. This is again in line with theory that suggests service use is facilitated by need.^20^

Finally, we found interactions between service use and child characteristics on the use of family disability services. In particular, families with males (vs females) were less likely to use FSCD services unless they had many years of severe coding. Next, when children with severe education codes were meeting expectations, service use did not differ by income. However, for families with a child below expectations, families in neighbourhoods of high SES were more likely to use services compared with those in low SES neighbourhoods. These findings replicate previous findings that social determinants of health affect family disability service use.^20^, ^21^ Such patterns are important considerations to service providers as they seek to facilitate equal access. Given that higher rates of disability are seen in lower‐income populations,^28^ further research is needed to understand how to facilitate the use of disability services in disadvantaged populations.

### Implications

This research poses implications to the service delivery of children with disabilities.

#### Eligibility

As family disability services and other disability services (i.e. intensive autism interventions, special education, etc.) require a diagnosis before services are offered, early identification is key to accessing these services. As one possible solution to this issue, screening tools may increase early identification;^29^ however, thoughts are mixed on screening tools and how to best use them.^30^, ^31^ In addition, relaxing the requirements for diagnosis and providing services when a demonstrable need is seen (i.e. the requirement for special education support) would increase access to services. This adds to the growing discussion for a need to shift from a ‘medical model of disability’ to models that focus on removing barriers to appropriate supports to optimize outcomes.

#### Referral

As the data show that families are more likely to use disability services when other service needs are involved, this speaks to the potential for referral to increase disability service access. However, to make the referral process stronger, it would be beneficial to have processes that enable easy identification of other potential services.^32^ Things such as education programs for practitioners and technology‐based resources that show potential services could help increase referral. In Alberta, the phone number 2–1–1 is one such resource that seeks to fill this role for referral to services, although this service is more focused on crisis diversion.^33^

#### Integrated service delivery

Owing to the complex needs of children with disabilities, integrated service delivery, where programs coordinate together to address needs, could increase access to services when they are needed. In Alberta, integration of services is a current discussion in movements such as the Regional Collaborative Service Delivery Program, where Alberta Education is working to increase conversations between provincial disability service providers,^34^ and the Integrated Hubs project, where municipal groups across Alberta are seeking to design one‐stop places to support children and young people with mental health issues.^35^ Furthermore, the data project that this paper draws upon had this very goal: to bring Alberta ministries together to provide coordinated support for children and young people.^36^

### Limitations

Despite the strength of using population‐based administrative data, this study had several limitations. First, administrative data suffer from limitations, including errors due to formatting and data entry issues, and difficultly in interpreting data patterns. In particular, not using family disability services may relate both to less need or to less access to services. As such, this research would benefit from future investigations (i.e. surveys and/or qualitative data) to provide more detail on families’ use of disability services. This research would help better understand the specific pathways of service use and the barriers families face. Second, while FSCD is one disability support service, other potential supports not represented in this study may be used by families (e.g. by health care providers, private providers, etc.). As such, our data understate the percentage of children getting any support beyond FSCD. For example, the lower percentages of family disability service uptake for children with emotional/behavioural issues may be partly explained by a lack of data on clinical psychologist and counselling service use. Future linkage between these programs would provide a more holistic picture of disability service access. Third, as missing data tended to be for lower SES and rural (vs urban) children, these findings may have limitations and generalizability issues for these populations. Furthermore, most of the missing data were seen for educational achievement in the model. As children with more severe disabilities may be less likely to take the Provincial Achievement Test and participation is not required, this may mean that the prevalence ratios for family disability service use are underreported (as this may exclude more severe cases). Finally, we cannot make causal interpretations based on these data. Future research is needed to determine what factors lead to disability service use.

## Conclusion

This study provides evidence of when and which families use disability services in the early years. The findings point to the potential benefit of discussions on access to services, including those on service eligibility, referral to services, and integration of services. Together the study can inform decisions on how to improve access to disability services among families with children with disabilities in the early years, to facilitate their full inclusion and participation.^1^

## Supporting information


**Appendix S1:** Categories of Special Education codes in the manuscript.

**Table S1:** Prevalence ratio for the association between child characteristics and service use, and the use of FSCD services for families with children with severe special education codes
