# Supplementary material for: Use of family disability service by families with young children with disabilities
Source: Dev Med Child Neurol. 2020 Jan 31;63(1):81–8. doi: 10.1111/dmcn.14478 (PMC7754115; doi:10.1111/dmcn.14478)
Supplement: Supplementary file 1 — Appendix S1: Categories of Special Education codes in the manuscript. [file DMCN-63-81-s002.doc]

**Supplementary Materials Appendix S1**

**Categories of Special Education codes in the manuscript**^1^ (derived from Alberta Education data)*:*

1. **Severe Emotional/Behavioural Disability** (*code 42*): “displays chronic, extreme and pervasive behaviours and requires close and constant adult supervision, high levels of structure, and other intensive support services in order to function in an educational setting. The behaviours significantly interfere with both the learning and safety of the student and other students.”
2. **Severe Delay Involving Language** (*code 47*): “has difficulty communicating with peers and/or adults because of a severe delay in expressive, receptive or total language.”
3. **Severe Physical/Medical Disability** (*code 44*): “has a medical diagnosis of a physical disability, specific neurological disorder or medical condition which creates a significant impact on the student’s ability to function in the school environment (note: some physical or medical disabilities have little or no impact upon the student’s/Early Childhood Services child’s ability to function in the school environment); and b) requires extensive adult assistance and modifications to the learning environment in order to benefit from schooling.” Includes Autism and Fetal Alcohol Spectrum Disorder diagnosed children.
4. **Severe Cognitive Disability** (*code 41*): “has severe delays in all or most areas of development, frequently has other associated disabilities including physical, sensory, medical and/or behavioural, and requires constant assistance and/or supervision in all areas of functioning including daily living skills.”
5. **Severe Multiple Disability** (*code 43*): “has two or more non-associated moderate to severe cognitive and/or physical disabilities that, in combination, result in the student functioning at a severe to profound level; and requires significant special programming, resources and/or therapeutic services.”

**Detailed definitions of covariates:**

*Covariates that were calculated over 2005/06 to 2010/11:*

1. Information on **sex** was provided for each child by participating programs. In the case of discrepancies between programs for sex, the most common value for an indicator was chosen. In the event of two or more most common values, the value for the indicator was chosen randomly from the most common values (derived from all available ministry data).
2. Postal codes were used to derive **City size** for the children, with a population of less than 10,000 being classified as *rural* and a population of 10,000 or more as *urban*. This definition is based on Statistics Canada definitions^2^. City size was defined as the average city type (derived from Alberta Health data).
3. **Socio-economic Status (SES)** captures the social and material environments in which youth lived. A youth was assigned a socio-economic status via an index based on the Statistics Canada dissemination area in which he or she resided^3^. Six indicators were included in the index: percent without a high school diploma, the employment rate, average income, percent of single families, percent of persons living alone, and percent of persons separated, divorced, or widowed. SES was defined by the average classification, with the bottom 40% of neighborhoods being coded as *low SES* and the top 60% of neighborhoods being coded as *high SES* (derived from Alberta Health data).
4. Alberta Education defines the population of **ESL** students as, “Children/students who require English as a Second Language program planning and instructional supports to achieve grade level learning expectations and reach their full potential”^4^. Students receiving ESL instruction must demonstrate challenges in English competencies, including reading, writing, speaking, and/or comprehension. In Alberta, ESL learners include students who have immigrated from countries outside of Canada. In addition, they include students who are Canadian-born, but whose first language is not English, such as students of Indigenous or Francophone descent^5^. ESL use was defined by at least one year of ESL designation (derived from Alberta Education data).
5. **High cost health service use**: Cost estimates were made based on the Canadian Institute for Health Information (CIHI) costs reported for physician visits (general practitioner or specialist), ambulatory care visits (emergency or other ambulatory care), and hospitalizations (by type of service). Estimated costs per visit were summed across all visits for each individual. High cost health users were those in the top 5% of estimated costs for their age groups and genders for at least one of the years (derived from Alberta Health data).
6. **Mental health service use** was defined by the presence or absence of a mental health diagnostic code in the primary ICD code in Alberta Health databases (Inpatient—Discharge Abstract Database, Ambulatory Care, and Practitioner Payments) in 2008/9 to 2010/11. Diagnostic codes included: depression, dissociative, somatoform, anxiety, schizophrenia, conduct, adjustment, bipolar, personality, substance use, self-harm, other psychoses, or other unspecified mental health conditions, based on Manitoba ICD code definitions (ICD-9: 291, 292, 295-298, 300, 301, 303-305, 307.9, 309, 311, 312; ICD-10: F1, F20-F22, F230, F232, F233, F238, F239, F25, F28-33, F340, F341, F380, F81, F412, F530, F40, F410, F411, F413, F418, F419, F42-45, F55, F60, F61, F68, F69, F91, F93, F989, F99, T36-40, R23, T421, T427, R43, T509, T58, T4, T50, X4, X6, X7, X80-84, Y10-12, Y16, Y17, Z502, Z503)^6^. Mental health service use is a proxy for presence of a mental health or other support need; some children with mental health needs may not have accessed mental health services in Alberta during the year, and some mental health service use may have occurred for children who did not have mental health needs (derived from Alberta Health data).
7. The **Family Support for Children with Disabilities (FSCD)** is an Alberta provincial program that provides a range of supports and services to families of children with disabilities. Family Support for Children with Disabilities works in partnership with eligible families to provide supports and services based on each child and family’s individual assessed needs. Services are meant to help strengthen families’ ability to promote their child’s healthy development and encourage their child’s participation in activities at home and in the community. Participation in the program is voluntary. FSCD use was defined by at least one year of use (derived from Community and Social service data).

*Covariate that was calculated in 2010/11 only:*

1. **Educational achievement** was computed by Alberta Education at the age of 8 years old (most children were in grade 3 in this period) using age, school type, special education codes, provincial achievement test scores, and current grade. Educational achievement was categorized as performing *below expectations* (i.e., having a moderate or higher intellectual, or severe multiple disability code, unsatisfactory provincial achievement (PAT) scores, or being behind a grade) or *meeting expectations* (i.e., not having a moderate or higher intellectual, or severe multiple disability code, having satisfactory or above provincial achievement (PAT) scores, or being on time in school based on their age) (derived from Alberta Education data).

**References**
