# Supplementary material for: Use of family disability service by families with young children with disabilities
Source: Dev Med Child Neurol. 2020 Jan 31;63(1):81–8. doi: 10.1111/dmcn.14478 (PMC7754115; doi:10.1111/dmcn.14478)
Supplement: Supplementary file 2 — Table S1: Prevalence ratio for the association between child characteristics and service use, and the use of FSCD services for families with children with severe special education codes [file DMCN-63-81-s001.pdf]

Table S1: The prevalence ratio (PR) for the association between child characteristics and service use, and the use of Family Support for Children with Disability (FSCD) services for families with children with severe special education codes. Reported interaction terms are significant. PRs reflect a comparison to the listed reference. Multivariable PRs control for other variables.

Child characteristics and service use	*Number that used FSCD Services*	*Unadjusted prevalence ratio*	*Multivariable prevalence ratio*
**Child characteristics**	N/Total (*%*)	PR(95% CI); *p-value*	PR(95% CI); *p-value*
Sex (Male)	453/1831 (*24.74%*)	.89(.77-1.02); = *0.09*	.76(.58-.98); < *0.04*
(*reference*: Female)	215/772 (*27.85%*)		
City Size (Rural)	123/520 (*23.65%*)	.90(.76-1.07); = *0.25*	.94(.80-1.10); = *0.43*
(*reference*: Urban)	545/2083 (*26.16%*)		
Socioeconomic status (Low SES)	292/1285 (*22.72%*)	.79(.69-.90); < *0.0005*	1.06(.88-1.27); = *0.55*
(*reference*: High SES)	366/1266 (*28.91%*)		
**Service use**			
ESL student (Yes)	51/273 (*18.68%*)	.71(.55-.91); < *0.008*	.89(.70-1.12); = *0.32*
(*reference*: No)	617/2330 (*26.48%*)		
Mental health service use (Used)	317/832 (*38.10%*)	1.92(1.69-2.18); < *0.0001*	3.06(2.34-4.00); < *0.0001*
(*reference*: Did not use)	351/1771 (*19.82%*)		
High cost health care (Yes)	478/1380 (*34.64%*)	2.23(1.92-2.59); < *0.0001*	1.57(1.37-1.80); < *0.0001*
(*reference*: No)	190/1223 (*15.54%*)		
Educational achievement (Below expect.)	303/767 (*39.50%*)	2.12(1.85-2.44); < *0.0001*	1.70(1.38-2.09); < *0.0001*
(*reference*: Meeting expectations)	278/1494 (*18.61%*)		
# of years education support (>4)	428/660 (*64.85%*)	5.25(4.61-5.99); < *0.0001*	5.50(4.10-7.37); < *0.0001*
(*reference*: 3 or less)	240/1943 (*12.35%*)		
**Interactions**			
# of years ed. support * Mental health			
>4 years ed. support & Used MH	188/290 (*64.83%*)	8.18(6.72-9.97); < *0.0001*	6.13(4.46-8.42); < *0.0001*
>4 years ed. support & No MH use	240/370 (*64.86%*)	8.19(6.75-9.94); < *0.0001*	5.50(4.10-7.37); < *0.0001*
3 or less ed. support & Mental health	129/542 (*23.80%*)	3.00(2.38-3.79); < *0.0001*	3.06(2.34-4.00); < *0.0001*
(*ref.*: 3 or less ed. support & No MH use)	111/1401 (*7.92%*)	1	1
# of years ed. support * Sex			
>4 years ed. support & Male	298/459 (*64.92%*)	4.36(3.54-5.37); < *0.0001*	5.99(4.51-7.95); < *0.0001*
>4 years ed. support & Female	130/201 (*64.68%*)	4.34(3.48-5.42); < *0.0001*	5.50(4.10-7.37); < *0.0001*
3 or less ed. support & Male	155/1372 (*11.30%*)	.76(.59-.97); < *0.03*	.76(.58-.98); < *0.04*
(*ref.*: 3 or less ed. support & Female)	85/571 (*14.89%*)	1	1
Educational achievement * Mental health			
Below expectations & Used MH	138/300 (*46.00%*)	3.56(2.92-4.35); < *0.0001*	3.87(2.90-5.18); < *0.0001*
Below expectations & No MH use	165/467 (*35.33%*)	2.74(2.24-3.34); < *0.0001*	1.70(1.38-2.09); < *0.0001*
Meeting expectations & Used MH	143/448 (*31.92%*)	2.47(2.01-3.04); < *0.0001*	3.06(2.34-4.00); < *0.0001*
(*ref.:* Meeting expectations & No MH use)	135/1046 (*12.91%*)	1	1
Educational achievement * SES			
Below expectations & Low SES	121/384 (*31.51%*)	1.65(1.34-2.03); < *0.0001*	1.31(1.04-1.64); < *0.03*
Below expectations & High SES	178/370 (*48.11%*)	2.52(2.10-3.02); < *0.0001*	1.70(1.38-2.09); < *0.0001*
Meeting expectations & Low SES	134/722 (*18.56%*)	.97(.79-1.20); = *0.79*	1.06(.88-1.27); = *0.55*
(*ref.:* Meeting expectations & High SES)	143/749 (*19.09%*)	1	1
